# Factors Associated with Use of Latex Condom-Compatible Lubricants by Men Who Have Sex with Men in India: Implications for HIV Prevention

**DOI:** 10.1155/2013/161085

**Published:** 2013-06-13

**Authors:** Shreena Ramanathan, Venkatesan Chakrapani, Lakshmi Ramakrishnan, Prabuddhagopal Goswami, Diwakar Yadav, Bitra George, Shrabanti Sen, Harikumar Rachakulla, Thilakavathi Subramanian, Ramesh S. Paranjape

**Affiliations:** ^1^FHI 360 India, H-5 (Ground Floor), Green Park Extension, New Delhi 110016, India; ^2^Centre for Sexuality and Health Research and Policy (C-SHaRP), 38 (Old No. 167), Rangarajapuram Main Road, Kodmbakkam, Chennai 600 024, India; ^3^National Institute of Nutrition (ICMR), Tarnaka, Jamai Osmania, Hyderabad 500007, India; ^4^National Institute of Epidemiology (ICMR), Second Main Road, TNHB, Ayapakkam, Chennai 600 077, India; ^5^National AIDS Research Institute (ICMR), T 71-1A/2, M.I.D.C., Telco Road, Bhosari, Pune 411 026, India

## Abstract

We examined the prevalence and type of rectal lubricants use and factors associated with exclusive use of latex-condom compatible lubricants (water-based lubricants) among men who have sex with men (MSM) using data from a large-scale cross-sectional survey conducted in 2009/10 in three Indian states. Using time-location cluster sampling, 3880 MSM were recruited from cruising sites. We used multiple logistic regression to assess the association between type of lubricants used and sociodemographic and programmatic indicators. Among those who reported using lubricants (64%) more than half (53%) exclusively used water-based lubricants, less than one-tenth used exclusively oil-based lubricants, and nearly 40% used both water-based and oil-based lubricants. Factors associated with exclusive use of water-based lubricants were exposure to HIV prevention interventions (AOR: 6.18, 95% CI 4.82 to 7.92) and kothi-identified MSM—feminine/anal-receptive (AOR: 2.56, 95% CI 2.12 to 3.10). Targeted HIV interventions among MSM need to promote and distribute latex condom-compatible lubricants for use during anal sex—irrespective of their presumed or stated sexual role in anal sex, and educate them not to use oil-based lubricants with condoms.

## 1. Introduction

Men who have sex with men (MSM) are a highly vulnerable population and substantially affected by the HIV epidemic worldwide [1]. In India, MSM have a very high HIV prevalence (7.4%) compared with the general population (0.31%). The National AIDS Control Organisation (NACO) has recognized MSM as a “key population” at risk for HIV and it funds targeted HIV interventions among MSM [2, 3]. Unprotected anal sex among MSM increases probability of transmission of HIV [4], but using water-based lubricants along with latex condoms can reduce HIV risk by reducing friction and condom damage [5]. Several studies [6–10] have documented that a significant proportion of MSM and hijras (male-to-female transgender people) in India use condoms inconsistently in anal sex acts and do not use latex condom compatible lubricants. 

In India, there is limited data on the use of lubricants along with condoms during anal sex by MSM. Understanding the extent and type of lubricants used by MSM will help policy-makers and program managers make evidence-informed decisions on promoting and distributing latex condom-compatible (water-based or silicone-based) lubricants along with condoms. We describe the prevalence and type of rectal lubricants use and factors associated with exclusive use of water-based lubricants among MSM in three southern states of India using data from a large cross-sectional bio-behavioural survey.

## 2. Methods

Data for the current analysis were drawn from the second in a series of cross-sectional surveys known as Integrated Behavioral and Biological Assessment (IBBA) conducted in 2009/10. That survey was part of evaluation of a large-scale HIV prevention program in three states in India: Andhra Pradesh, Tamil Nadu, and Maharashtra. The detailed survey methodology is provided elsewhere [11, 12]. MSM participants were recruited using time-location cluster sampling from cruising sites such as parks and local train stations. In Andhra Pradesh and Maharashtra, the inclusion criteria were self-identified MSM who were 18 years of age or older and who had any type of sex (oral, anal, or manual) with another man in the past month. In Tamil Nadu, the inclusion criteria were similar, except that the type of sex was limited to anal sex with another man in the past month. Written informed consent was obtained from all participants. Behavioral data were collected using a structured interviewer-administered questionnaire, and blood and urine samples were tested for HIV and sexually transmitted infections (STIs). The study protocol was approved by the local ethics committees of the implementing institutes of Indian Council of Medical Research (National AIDS Research Institute, Pune; National Institute of Nutrition, Hyderabad, and National Institute of Epidemiology, Chennai) and Protection of Human Subjects Committee of FHI 360. 

Lubricant use was assessed by the following question: have you ever used a lubricant while having anal sex? Response options were yes, no, or no answer. Type of lubricants used in the past year was assessed by a multiple response question where up to six responses could be recorded. The response choices included baby oil, butter, cooking oil, coconut oil, hand moisturising lotion, KY Jelly, Vaseline, and saliva. As the analytic focus was to examine the factors associated with exclusive use of water-based lubricants, based on the combination of responses received, we created three sets of variables in relation to the type of lubricants used. One set was created for the descriptive analysis and it had three categories: (a) exclusive oil-based lubricant users (those using baby oil, butter, cooking oil, coconut oil, and hand moisturising lotion), (b) exclusive water-based lubricant users (those using KY Jelly, a famous brand of water based lubricants) also referred to as latex condom compatible lubricant users in the paper, and (c) mixed users (who reported using oil-based or water-based lubricants, or saliva). Two other sets were created for multivariate analysis each having two categories: exclusive water-based lubricant users and mixed users; and exclusive water-based lubricant users and non-users. 

Exposure to HIV interventions was measured using a composite indicator created from three questions that assessed whether the participants ever visited clinics operated by nongovernmental organizations, ever received condoms from peer educators or outreach workers, and were ever contacted by peer educator or outreach workers. Those who reported receiving any one of these three services were considered to be “exposed to any HIV prevention intervention.” Similarly, those MSM who tested positive in blood or urine samples for either one of the three STIs—gonorrhoea, chlamydiasis, and syphilis—were categorized as “having any STI.” Syphilis was screened by Rapid Plasma Reagin (RPR) test and confirmed by Treponema Pallidum Hemagglutination Assay (TPHA). Nucleic-acid amplification (Gen-Probe APTIMA Combo 2—Gen-Probe Inc., San Diego, CA, USA) tests on urine samples were conducted for chlamydial infection and gonorrhoea. Bivariate associations between type of lubricant used and independent variables (related to sociodemographics and exposure to HIV interventions) were assessed by Chi-square tests, and logistic regression was used to identify correlates of exclusive use of water-based lubricants versus non-users or mixed users. All analyses were conducted in Stata-11 (Stata Corporation, College Station, TX, USA). 

When conducting bivariate and logistic regression analyses, to aid in analysis and interpretation, variables which initially had three or more categories were collapsed into two meaningful categories, especially if there were very low frequencies in certain categories of those variables. Therefore, age distribution was categorised into “≤25 years” and “above 25 years,” educational status was categorized as “less than higher secondary” and “higher secondary and above,” and marital status was categorized as “never married” and “ever married.” For occupation, we categorized respondents into two groups: “manual laborers” (agricultural/nonagricultural laborers) and another group that included “students, unemployed, and white-collar workers.” While the proportion of MSM with different self-identities are provided in Table 1, for logistic regression, we categorized self-identity of MSM as kothis and “nonkothis,” the latter including identities such as panthi, double decker, and bisexual-identified MSM. This was because, in general, kothis are relatively visible, well organized, and more accessible to interventions when compared with other subgroups of MSM [13]. Similarly, for alcohol consumption, we created two groups: drinkers (everyday, at least once a week, and less than once a week) and nondrinkers (never in life and never in the past month), as alcohol use in general has been shown to influence condom use among Indian MSM [14]. 

## 3. Results

Of the 3,880 MSM who were interviewed, a majority reported using lubricants (64%, *n* = 2487/3872; 60.4% in Andhra Pradesh, 42.4% in Maharashtra, and 76.6% in Tamil Nadu) and more than half of these (53%, *n* = 1325/2485) exclusively used water-based lubricants. Less than one-tenth of users (7.6%, *n* = 191/2485) reported exclusive use of oil-based lubricants and nearly 40% (*n* = 969/2485) of these MSM were mixed users (using both water-based and oil-based lubricants) (see Table 1).

Characteristics of lubricant non-users (NU) and exclusive water-based lubricant users (EWU) are presented in Table 2. When compared with non-users, EWU were more likely to be residents of Tamil Nadu (EWU-58.4%, NU-27.2%, *P* = 0.000), exposed to HIV prevention programs (EWU-93%, NU-60%; *P* = 0.000), self-identify as kothis (EWU-71%, NU-38%; *P* = 0.000), consume alcohol (EWU-63%, NU-59%; *P* = 0.023), aged above 25 years (EWU-55%, NU-48%; *P* = 0.000), and have HIV (EWU-12.9%, NU-9.8%; *P* = 0.013) and STIs (EWU-7.8%, NU-5.7%; *P* = 0.032) (see Table 2). 

Results of the multivariate analysis are shown in Table 3. Characteristics associated with increased odds for exclusive use of water-based lubricants taking non-users as the reference group were exposure to HIV prevention interventions (AOR: 6.18, 95% CI 4.82–7.92), self-identity as kothis (AOR: 2.56, 95% CI 2.12–3.10), and consumption of alcohol (AOR: 1.28, 95% CI 1.07–1.53). When adjusted for sociodemographic and other contextual factors such as exposure and consumption of alcohol, HIV and STI prevalence did not have a statistically significant association. We obtained similar results (details are not shown) when we had mixed users as the reference group instead of non-users except that those who were HIV-positive were at decreased odds (AOR: 0.75, 95% CI 0.58–0.95) for exclusive use of water-based lubricants when compared with those who were HIV negative. 

## 4. Discussion

Our analysis has provided program-relevant information on the extent and type of rectal lubricants use among Indian MSM and the factors that might facilitate the use of water-based lubricants. More than half of the lubricant users reported exclusively using water-based lubricants in the past year, and exposure to HIV prevention intervention was found to be a significant associated factor. During the period between Round 1 and 2 of IBBA (i.e., before and during this survey), HIV interventions among MSM in the study sites promoted use of water-based lubricants along with condoms, and in some study sites (such as Mumbai and Hyderabad), water-based lubricants were distributed freely or provided at a subsidized cost through social marketing [15, 16]. Thus, our finding indicates that Indian MSM are willing to use water-based lubricants, if information is provided on condom-compatible (water-based or silicone-based) lubricants and access to lubricants is facilitated through interventions. 

Another key finding that about one-third of the lubricant users who reported using both water-based and oil-based lubricants could mean that many MSM may not be aware that oil-based lubricants should not be used with condoms [17–19]. This is despite the fact that the operational guidelines (2007–2012) of the National AIDS Control Organisation (NACO) for targeted HIV interventions for MSM recommend use of only water-based lubricants along with condoms during anal sex [20]. It is also possible that many MSM might have very limited access to water-based lubricants, given that they are expensive, and the free or subsidised lubricants distributed in targeted HIV interventions for MSM would have barely met the needs of all MSM [9, 21]. If oil-based lubricants are used along with condoms during anal sex, then the chances of condom breakage are higher thereby increasing the risk of HIV transmission. This might explain why we found a statistically significant association between being HIV-positive and using both water- and oil-based lubricants. 

The finding that kothi-identified MSM are more likely to report using water-based lubricants when compared with other subgroups (like double-decker—both anal-insertive and anal-receptive or panthi—anal-insertive) could be because HIV interventions among MSM in India are reported to reach a higher proportion of kothis [13] and thus more likely to have access to water-based lubricants or information about the proper use of lubricants [21]. This also indicates the need to intensify efforts to promote water-based lubricants among other subgroups of MSM such as double-deckers and panthis. 

Alcohol consumption was significantly associated with exclusive use of water-based lubricants. This finding is counter-intuitive as alcohol consumption is usually associated with HIV-related sexual risk behaviors such as multiple sex partners and inconsistent condom use [22, 23]. As we did not collect any sexual event-level data on alcohol use, condom use, and lubricant use, we could not provide plausible reasons for the association between alcohol use and water-based lubricant use in this sample. 

A major gap in this analysis is the lack of information on the use of water-based lubricants in last anal sex and consistency of lubricant use in a recent timeframe (past month) and whether type and consistency of lubricants use differ according to type of partner (regular, casual, or paid). Factors associated with lubricant use among hijras or transgender people may be quite different from MSM, but our study did not have adequate sample size of hijras for a separate comparative analysis. As we did not have specific questions on the use of lubricants along with condoms, for example, use of lubricant along with a condom in the last anal sex episode, it is possible that lubricants could have been used without condoms. However, almost all the persons (>95%) who reported using lubricants also reported using condoms (although inconsistently) in anal sex, making it less likely that lubricants could have been used without condoms in this sample. Future studies need to address these gaps. 

Despite these limitations, the current analysis provides important program-relevant information on rectal lubricants use among MSM in India, which has not been available until now. The familiarity and widespread use of water-based lubricants among MSM means that the national HIV program, through its targeted HIV interventions among MSM, has an opportunity to provide information on the effects of oil-based lubricants on condom slippage and breakage and to promote and distribute latex condom-compatible lubricants along with condoms during anal sex. Intensifying the efforts to reach all subgroups of MSM (kothis, panthis, double-deckers, and bisexuals) through targeted interventions and promoting latex condom-compatible lubricant use among them, irrespective of their presumed or stated sexual role (receptive or insertive or both) in anal sex, will help in increasing the use of latex condom-compatible lubricants during anal sex. 

## Figures and Tables

**Table 1:** Sociodemographic characteristics of men who have sex with men in Maharashtra, Tamil Nadu, and Andhra Pradesh (Round 2 IBBA: 2009-2010).

Variables	Round 2 IBBA (2009-2010) (*n* = 3880)
Age (years) (mean = 27)	
18–25	47.5 (1846)
26–35	38.8 (1508)
36–45	10.7 (415)
46 and above	2.8 (111)
Educational attainment	
Illiterate	12.2 (495)
Up to 8th grade	24.1 (936)
9th to 12th grade	49.8 (1933)
Any college education	13.3 (516)
Marital status	
Never married	70.6 (2741)
Currently married	28.2 (1097)
Widowed/divorced/others	1.08 (42)
Occupational status	
Unemployed/student	10.9 (426)
Self-employed	20.4 (794)
Nonagricultural labor	25.2 (981)
Govt./Pvt. employee	24.9 (969)
Others	18.3 (710)
Self-identity	
Kothi^#^	54.6 (2120)
Panthi^##^	13.3 (516)
Double Decker^##^	11.4 (446)
Bisexual^##^	18.1 (703)
Hijra	2.4 (95)
Alcohol consumption (past month)	
Everyday	6.1 (237)
At least once a week	33.4 (1298)
Less than once a week	20.1 (783)
Not in the past month	6.6 (256)
Never consumed	33.5 (1301)
Reported condom breakage (past month)	
No	83.1 (3164)
Yes	16.8 (642)
Received condoms from HIV program	
No	3.0 (93)
Yes	96.9 (2937)
Exposure to program (past one year)	
No	23.0 (895)
Yes	76.9 (2985)
Any STI	
Negative	93.1 (3615)
Positive	6.8 (265)
HIV	
Negative	87.4 (3392)
Positive	12.5 (488)
Use of any type of lubricant	
No	35.7 (1385)
Yes	64.2 (2487)
Type of lubricant used (past one year)	
Exclusively water-based∗	53.3 (1325)
Exclusively oil-based∗∗	7.6 (191)
Mixed users∗∗∗	38.9 (969)
Use of any type of lubricant by state	
Andhra Pradesh	60.4 (969/1608)
Maharashtra	42.4 (276/652)
Tamil Nadu	76.6 (1242/1620)

Totals may not add up in all variables because of exclusion of missing data and “do not know” responses.

^#^Kothi—anal-receptive; ^##^Panthi—anal-insertive; Double Decker—anal-insertive and anal-receptive.

∗MSM who reported using only latex condom-compatible lubricants in the past year.

∗∗MSM who reported using oil-based products such as baby oil, butter, cooking oil, moisturising hand lotion, and vaseline in the past year.

∗∗∗MSM who reported using water-based and oil-based lubricants in the past year.

**Table 2:** Characteristics of non-users and exclusive water-based lubricant users in Maharashtra, Tamil Nadu, and Andhra Pradesh (Round 2 IBBA: 2009-2010).

Variables	Non-users (NU) (*n* = 1385)	Exclusive water-based lubricants users (EWU) (*n* = 1325)	*P* value
State			
Andhra Pradesh	45.7 (633)	34.4 (456)	0.000
Maharashtra	27.0 (374)	7.1 (95)	
Tamil Nadu	27.2 (378)	58.4 (774)	
Age			
≤25	52.2 (724)	44.5 (590)	0.000
Above 25	47.7 (661)	55.4 (735)	
Educational attainment			
Less than higher secondary	34.6 (480)	37.6 (398)	0.104
Higher secondary and above status	65.3 (905)	62.3 (658)	
Marital status			
Never married	68.8 (953)	74.1 (982)	0.002
Ever married	31.1 (432)	25.8 (343)	
Occupational status			
Student/unemployed/white-collar worker	77.0 (1061)	69.3 (675)	0.000
Manual laborer	22.9 (316)	30.6 (377)	
Self-identity			
Kothi	38.1 (529)	71.4 (947)	0.000
Panthi	25.0 (347)	4.4 (59)	
Double Decker	10.4 (145)	8.6 (114)	
Bisexual	23.1 (321)	13.0 (173)	
Hijra	3.1 (43)	2.4 (32)	
Alcohol consumption			
No	41.3 (572)	37.1 (492)	0.023
Yes	58.6 (810)	62.8 (833)	
Exposure to HIV program			
No	40.2 (557)	6.9 (92)	0.000
Yes	59.7 (828)	93.0 (1233)	
Any STI			
Negative	94.2 (1305)	92.1 (1221)	0.032
Positive	5.7 (80)	7.8 (104)	
HIV			
Negative	90.1 (1248)	87.0 (1154)	0.013
Positive	9.8 (137)	12.9 (171)	

**Table 3:** Association between exclusive use of water-based lubricants by MSM and exposure to HIV prevention interventions in Maharashtra, Tamil Nadu, and Andhra Pradesh (Round 2 IBBA: 2009-2010).

Variables	Exclusive use of water-based lubricants	
	Crude odds ratio (CI)	Adjusted odds ratio (CI)
Exposure to HIV program		
No	1.00	1.00
Yes	9.01 (7.10–11.43)∗∗	6.18 (4.82–7.92)∗∗
Age (years)		
≤25	1.00	1.00
Above 25	1.36 (1.17–1.58)∗∗	1.05 (0.86–1.28)
Educational attainment		
Less than higher secondary	1.00	1.00
Higher secondary and above	0.87 (0.75–1.02)	0.95 (0.79–1.15)
Marital status		
Never married	1.00	1.00
Ever married	0.77 (0.65–0.91)∗∗	0.94 (0.75–1.18)
Occupational status		
Student/unemployed/white-collar worker	1.00	1.00
Manual laborer	1.48 (1.25–1.76)∗∗	1.18 (0.97–1.44)
Self-identity		
Nonkothi^#^	1.00	1.00
Kothi (anal-receptive)	4.05 (3.45–4.76)∗∗	2.56 (2.12–3.10)∗∗
Alcohol consumption		
No	1.00	1.00
Yes	1.19 (1.02–1.39)∗	1.28 (1.07–1.53)∗∗
Any STI		
Negative	1.00	1.00
Positive	1.38 (1.02–1.87)∗	1.23 (0.86–1.74)
HIV		
Negative	1.00	1.00
Positive	1.34 (1.06–1.71)∗	1.20 (0.91–1.58)

Two cases that reported using lubricants but did not specify the type were excluded from the analysis.

^#^“Nonkothi” includes MSM who identified as panthi, double decker, bisexual, and hijra.

∗ *P* < 0.05, ∗∗*P* < 0.001.
